# TEFTOM: A Promising General Trauma Expectation/Outcome Measure—Results of a Validation Study on Pan-American Ankle and Distal Tibia Trauma Patients

**DOI:** 10.1155/2013/801784

**Published:** 2013-02-28

**Authors:** Michael Suk, Monica Daigl, Richard E. Buckley, Cleber A. J. Paccola, Dean G. Lorich, David L. Helfet, Beate Hanson

**Affiliations:** ^1^Orthopaedic Trauma Service, College of Medicine Jacksonville, University of Florida, 655 West Eight St, 2nd Floor ACC, Jacksonville, FL 32209, USA; ^2^Clinical Investigation and Documentation, AO Foundation, Stettbachstrasse 6, 8600 Duebendorf, Switzerland; ^3^Department of Surgery, Foothills Medical Centre, University of Calgary, AC 144A, 1403 29th Street NW, Calgary, AB, Canada T2N 2T9; ^4^Department of Orthopaedics and Trauma Surgery, Ribeirão Preto School of Medicine, São Paulo University, São Paolo, Brazil; ^5^Orthopaedic Trauma Service, New York Presbyterian University Hospital of Columbia and Cornell, 525 East 68th Street, New York, NY 10065-4870, USA; ^6^Orthopaedic Trauma Service, Hospital for Special Surgery, 535 East 70th Street, New York, NY 10021-4898, USA

## Abstract

*Background*. In orthopedics, there is no instrument specifically designed to assess patients' expectations of their final surgery outcome in general trauma populations. We developed the Trauma Expectation Factor Trauma Outcome Measure (TEFTOM) to investigate the fulfilment of patients' expectations one year after surgery as a measure of general trauma surgical outcomes. The aim of this paper was to assess the psychometric characteristics of this new general trauma outcome measure. *Methods*. The questionnaire was tested in 201 ankle and distal tibia fracture patients scheduled for surgery. Patients were followed up for twelve months. The TEFTOM questionnaire was evaluated for its criterion validity, internal consistency, reproducibility, and responsiveness. *Results*. TOM showed good criterion validity against the American Academy of Orthopaedic Surgeons Foot and Ankle Scale (Pearson's correlation coefficient = 0.69–0.77). Internal consistency was acceptable for TEF (Cronbach's alpha = 0.65–0.76) and excellent for TOM (Cronbach's alpha = 0.76–0.85). Reproducibility was moderate to very good (intraclass coefficient correlation (ICC) ≥0.67) for TEF and very good (ICC ≥0.92) for TOM. TOM also proved to be responsive to changes in patients' condition over time (Wald test; *P* < 0.001). *Conclusions*. TEFTOM is a promising tool for measuring general trauma outcomes in terms of patients' expectation fulfilment that proved to be valid, internally consistent, reproducible, and responsive to change.

## 1. Background

Ministries of health and healthcare providers from various countries are shifting their focus from clinical processes to outcomes, that is, concentrating on the quality rather than quantity of healthcare [1–3]. How much hospitals get paid for a procedure may soon depend in part on such measurements of outcome [4]. Furthermore, patient-reported outcomes in addition to clinical quality indicators [5] are becoming more popular [6–8]. It is therefore essential to have valid and reliable outcome measures tailored to each field of application.

Generic measures have been proposed for chronic disease or injury [9, 10] to assess provider performance in improving the patient's condition. However, these measures are inappropriate for evaluating trauma outcomes, as no reliable baseline function measurements are available for trauma patients. While a baseline measurement of condition is neither possible nor helpful, a baseline expectation measurement of condition is achievable. Discovering this information may be the key to a new outcome paradigm that considers patients' expectations in light of their final personal outcome. Mondloch et al. recommended working toward a core set of reliable and valid measures of patients' expectations, bearing in mind that “the best prediction of outcome would be an expectancy measure whose domain of behaviour matches that of the outcome” [11].

In orthopaedics, the relationship between patients' expectations and outcomes has been evaluated in various trauma conditions [12–15]. This work led to the development of surgery expectation surveys for specific trauma conditions. In the authors' opinion, these instruments can provide a way to learn about patients' perspectives, thus providing the surgeon with a template to guide formal discussion about realistic and unrealistic goals and a prospective record that can be used jointly by the surgeon and the patient postoperatively to assess the surgical outcome. 

A systematic review of over 300 musculoskeletal outcome measures currently available for clinical and research purposes [16] revealed that there was no instrument specifically designed to assess patients' expectations for their final surgery outcome in general trauma populations scheduled for surgery. 

We developed a patient self-rating instrument, the Trauma Expectation Factor Trauma Outcomes Measure (TEFTOM), as a standardised tool to investigate the fulfilment of patients' expectations as a measure of general trauma surgical outcomes. 

The TEFTOM questionnaire consists of two portions: the TEF portion is administered preoperatively to assess patients' expectations following consultation with the orthopaedic surgeon, whereas the TOM portion is administered one year after surgery to assess surgery outcome and quantify the extent to which patients' expectations have been fulfilled. The questionnaire was evaluated in a prospective, multicentre study in ankle and distal tibia fracture patients. The aim of this paper is to describe the assessment of the psychometric characteristics (criterion validity, internal consistency, reproducibility, and responsiveness) of this new and promising general trauma outcome measure.

## 2. Methods

### 2.1. Development of the TEFTOM Questionnaire

Our research team of trauma surgeons led by the primary (MS) and senior authors (BH) performed a systematic review of over 300 musculoskeletal outcome measures currently available for clinical and research purposes [16]. Based on this meticulous screening of all available tools, it was found essential to develop a generic “core set” tool, which has not yet been considered in the general orthopaedic trauma population to assess the reflection of patient expectation on predicting their final outcome after trauma; this instrument was required to be clinician and patient friendly as well as parsimonious. This was the basis for developing the TEFTOM tool, which was assembled from adaptations of a well-established “core set” questionnaire developed for spine/lower back pain research that has already been proven to be valid, reliable, and responsive [17]. The ten items of the TEFTOM questionnaire were selected to cover the five essential domains proposed by Deyo et al. [18] and Bombardier et al. [19] of pain, physical function, disability, injury satisfaction, and overall satisfaction.

The generated 10-item TEFTOM tool was pretested on 20 patients with a distal tibia fracture. Patients were asked to provide feedback on format, comprehensibility, and content. No ambiguous, inappropriate, or unclear questions were reported. None of the questionnaires returned had blank items. Patients were also asked to report whether they felt that some important issues were missing. None of them reported any concern. Based on this feedback and review by the study team, the psychometric assessment of the questionnaire was initiated.

The TEFTOM instrument comprises two parts of 10 items each, which address the five mentioned domains (see the Appendices A and B). The most important aspect of this measure is that each question can be easily adapted to assess either expectation or outcome. The ten items are individually scored using a 5-point rating scale (from 0 to 4). An overall score ranging from 0 (lowest expectation/outcome) to 40 (highest expectation/outcome) can be easily calculated, whereby the point systems for items from 1 to 7 are first reversed, and a raw score is calculated by summing up all 10 items. 

The tool was developed to be self-administered. The reference period for expectations and outcomes was set to one year after surgery. Response options were chosen as ordinal rating scales to offer a clear distinction between choices and were designed, so that each item would have the same weight. 

While these questions were developed in English, they were deemed suitable for meaningful translation into Portuguese by clinicians in Brazil for one of our international study centres. 

### 2.2. TEFTOM Validation Study

A prospective multicentre study was conducted to evaluate the performance of the TEFTOM questionnaire. Although the target population of the TEFTOM tool is general trauma patients, we performed our validation study in patients with ankle and distal tibia fracture scheduled for surgery.

The main inclusion criterion was an isolated ankle or distal tibia fracture in patients aged 18 years or older who had provided written informed consent to participate in the study. The operative procedure was required to be performed within four weeks after the injury. 

Exclusion criteria included previous internal fixation surgery of the injured ankle and medical conditions that affect bone union, such as metastatic cancer and metabolic bone disease. Polytraumatised patients were also excluded. Other excluded patients were those with severe dementia or other severe mental health problems that would preclude them from completing study questionnaires, those who knew that they would be unable to attend all scheduled study-related followup visits, those participating in other clinical trials of a drug or device, and prisoners. 

Five participating clinics in Brazil, Canada, and the United States (USA) were involved. Institutional review board approval was obtained at each centre, and informed consent was obtained from each participant included in the study.

### 2.3. Followup Examinations

Although the questionnaire is designed to be self-administered before surgery and then one year after surgery, for the questionnaire validation study patients were actively followed up at several time points during one year. Patients underwent a physical assessment and personal interview by the treating surgeon to complete the TEFTOM questionnaire as follows: the TEF portion was administered before and immediately after surgery, and then at two weeks, six weeks, three months, and six months after surgery, while the TOM portion was administered at three, six, and twelve months after surgery. Additional retesting of the TEF and TOM portions was performed at the same time points in those patients who consented to participate in a test-retest reliability study. In addition, patients completed the American Academy of Orthopaedic Surgeons Foot and Ankle Scale (AAOS), the Foot and Ankle Outcome Score (FAOS), and the Short Form-36 Health Survey (SF-36) questionnaires at the same time points scheduled for the TOM portion of the questionnaire.

### 2.4. Instruments

AAOS comprises 20 questions that cover four subscales targeting pain, function, stiffness and swelling, and giving way [20] and was used to assess criterion validity of the TOM portion of the questionnaire. FAOS is a disease-specific measure for ankle instability consisting of 42 items, which measure five subscales: pain, other symptoms, activities of daily living, sports and recreation, and quality of life [21]. SF-36 is a comprehensive questionnaire on general patient health and well being [22]. All three instruments were self-administered. For use at the Brazilian site, the instruments were translated into Portuguese.

### 2.5. Patient Demographics and Characteristics

A sample size of 200 patients was planned for this investigation. A total of 204 patients were recruited between October 2006 and January 2009; the entire study period spanned from when the first patient was recruited in October 2006 to the last patient's 1-year followup visit in February 2010. Two patients declined to participate in the study before the surgery was performed, and another patient who had surgery 45 days after the trauma event was excluded from the study. Four additional patients did not satisfy the isolated ankle or distal tibia fracture inclusion criterion but received a waiver from the orthopaedic surgeon who rated their secondary fractures as unlikely to interfere with their perception of the ankle/distal tibia fracture. Therefore, a total of 201 patients were included in the data analysis. Patient baseline sociodemographic characteristics are presented in Table 1.

**Figure 1:**
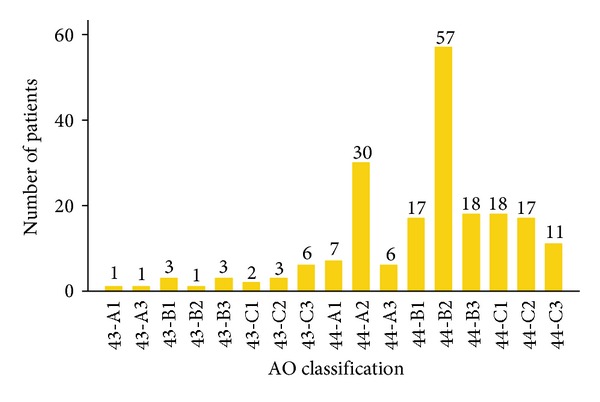
Distribution of AO classified fractures.

Included in the study were 181 ankle fractures (90%)—AO Foundation and Orthopaedic Trauma Association (AO/OTA) Type 44—and 20 distal tibia fractures (10%)—AO/OTA Type 43 (Figure 1) [23]. Seven fractures (3.5%) were open, and 91 (45.3%), 80 (39.8%), and 23 (11.4%) were classified as Grade 0, I, and II/III closed injuries according to Tscherne and Oestern [24].

### 2.6. Operative Data

Patients underwent the normal standard of care at each institution with respect to surgery, postoperative care, and rehabilitation protocols. Half of the patients were treated within five days after the trauma (range: 5–27 days). The median duration of the surgery was 80 minutes (interquartile range: 43–110 minutes). The primary surgical intervention for 177 (88%) patients was open reduction with internal fixation only, followed by 20 (10%) who initially received external fixation prior to the open reduction and internal fixation procedure; only 4 (2%) underwent external (*n* = 1) or percutaneous (*n* = 3) fixation. 

One hundred and thirty-four (67%) fractures were treated by chief surgeons who had previous experience with over 30 procedures using the same surgical technique. In 194 patients (97%), the surgeons were satisfied with the final immediate postoperative outcome. In 7 patients (3%) the surgeons were not satisfied with the final surgical outcome: in 6 patients surgeons reported failure to achieve anatomic reduction, and in 1 patient surgeons reported severe comminution of the medial malleolus.

### 2.7. Statistical Analysis

Patients recruited in the study were considered for the analysis up to the point of their last study visit. Patient recruitment and followup visit records obtained within the following time windows were analysed: 2 weeks before surgery, immediate postoperative period within 5 days after surgery, 2 weeks ±7 days, 6 weeks ±14 days, 3 months ±30 days, 6 months ±45 days, and the final period of 10 to 20 months based on the assumption that the 2-year outcome would resemble what achieved at 1 year. Statistical analyses were performed using Intercooled Stata Version 11 statistical software (Stata Corp, College Station, TX). 

In order to estimate averages for the scores of interest at each time point we used mixed-effects linear regression with random patient effects to account for repeated (longitudinal) measurements on the same patient. Likelihood ratio tests were performed to verify trends over time. 

The TEFTOM questionnaire was evaluated for its criterion validity, internal consistency, reproducibility, and responsiveness. 

### 2.8. Criterion Validity

The criterion validity of the TOM element of the questionnaire was assessed by means of Pearson's correlation coefficient against the AAOS, which is a gold standard measure of the condition of interest. A coefficient between 0.8 and 1 indicates “very good or strong” correlation. Correlations of the TOM score with FAOS and SF-36 were also investigated. 

### 2.9. Internal Consistency

Internal consistency was assessed for TEFTOM using Cronbach's alpha and is calculated from the pairwise correlations between the questionnaire items. This measure ranges from zero to one, where the following benchmarks were considered: 0.6-0.7 acceptable consistency, 0.7-0.8 satisfactory consistency, and 0.8 or higher very good consistency [25]. Values of 0.95 or higher are not necessarily desirable and most often indicate the redundancy of the items. Therefore, the goal of designing a reliable instrument in terms of internal consistency is to include similar items that are related (i.e., internally consistent), yet individually provide some unique information. 

### 2.10. Test-Retest Reliability (Reproducibility)

The reproducibility of the TEFTOM questionnaire was assessed by means of calculating the intraclass correlation coefficient (ICC) at different time points. ICC measures the agreement between scores obtained by the same subject separated by a short period of time. An ICC of 1 indicates absolute agreement and is obtained after every patient scores exactly the same when the tool is readministered on a second occasion. 

### 2.11. Responsiveness (Sensitivity to Change)

A multilevel mixed-effects linear regression was also used to test responsiveness of the TOM score. If the TOM portion was responsive to change, a significant increase in the measured outcome would be observed between the 3- and 6-/12-month followup evaluations based on the Wald test. 

## 3. Results

**Figure 2:**
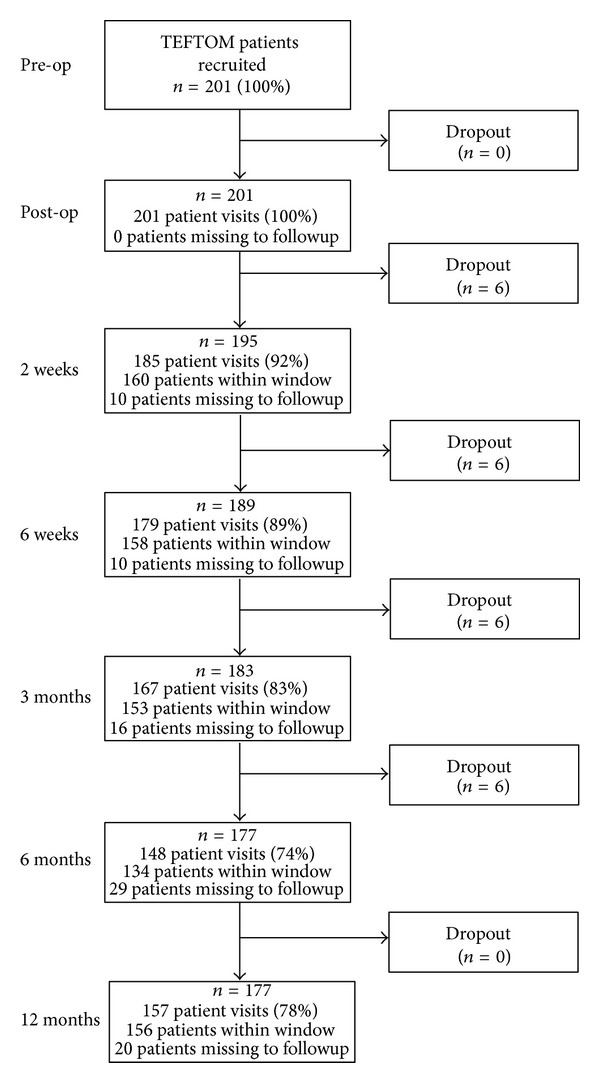
Patient recruitment and followup flow chart.

The patient recruitment and followup flow chart is provided in Figure 2. Followup rates were consistently at or above 74% (148/201). 

Average patient expectations as measured with the TEF portion of the questionnaire ranged from 33.9 to 35.3 points over the 6-month period (Table 2), yet patients were not consistent in reporting their expectations over time (Likelihood Ratio Test; *P* < 0.001).

All outcome measures improved over time between the 3-month and 1-year evaluations (Table 2). The 1-year mean TOM score was less prone to a ceiling effect (i.e., reaching the uppermost end of the scale) than the AAOS and FAOS “activities of daily living” dimension. In fact, the average 1-year TOM score only lay at 20% below the upper limit of the scale, whilst the mean AAOS score was 11% below the upper limit of the AAOS scale. While 10% of the patients had reached the maximum score of 40 on the TOM scale at 1-year followup, 14% of the patients had an AAOS score of 100 at 1 year. 

### 3.1. Criterion Validity

**Figure 3:**
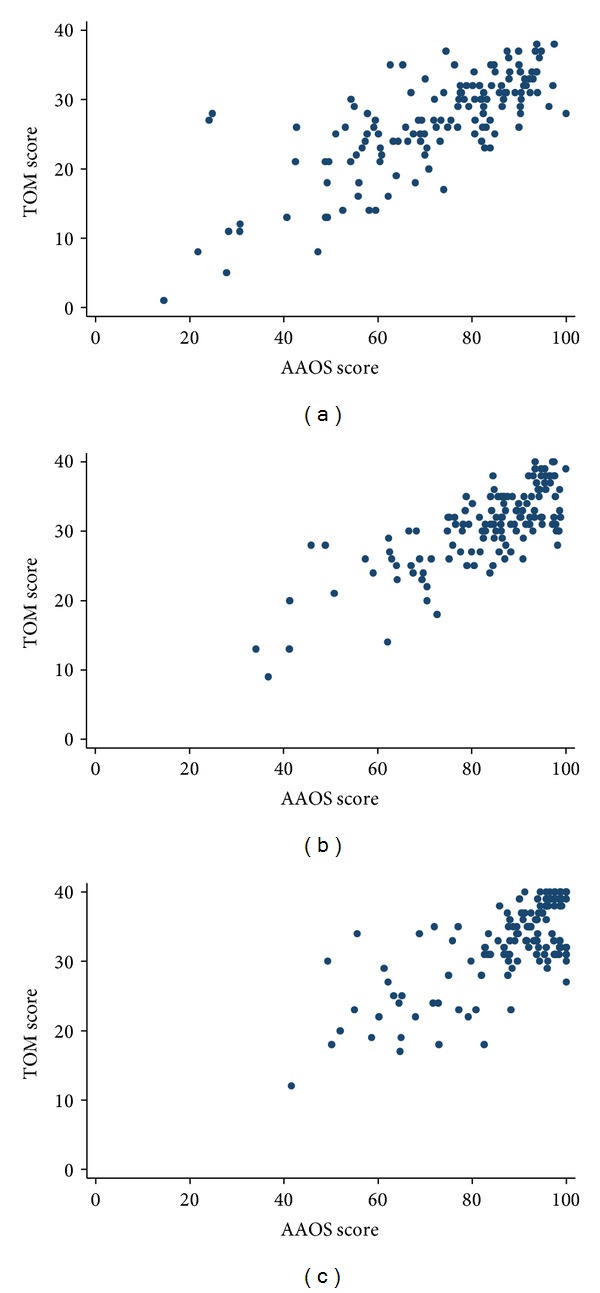
Correlation between the TOM questionnaire and AAOS tool at (a) 3 months, (b) 6 months, and (c) 1 year.

The TOM questionnaire showed good criterion validity with the AAOS (Figure 3). Pearson's correlation coefficients ranged from 0.69 to 0.77 between the 3-month and 1-year testing period (Table 3). Correlation coefficients for validation against the five FAOS dimensions and the SF-36 Physical Component Summary were all equal to or lower than 0.7. In addition, correlations between the TOM portion and the unweighted average of the three SF-36 physical subscales ranged from 0.66 to 0.73 between the 3-month and 1-year testing period.

### 3.2. Internal Consistency

Internal consistency of the TEF portion proved to be acceptable, with Cronbach's alpha ranging from 0.65 to 0.76 over the 6-month testing period (Table 4). Internal consistency of the TOM tool proved to be excellent, with Cronbach's alpha ranging from 0.85 at three months to 0.76 at twelve months (Table 5). 

### 3.3. Test-Retest Reliability (Reproducibility)

A total of 80 patients consented to participate in the reliability assessment of the TEF tool. These patients were contacted by phone on average one day after their clinical examination, that is, one day after the day on which the form was completed (range: 0–14 days; median: 0). At all postoperative time points, TEF reliability was moderate to very good, with ICCs between 0.67 and 0.94 (Table 4). Sixty-two patients participated in the TOM reliability assessment. As for the TOM questionnaire, patients were contacted by phone on average one day after their clinical examination (range: 0–14 days; median: 0). The ICCs ranged between 0.92 at three months and 0.96 at twelve months (Table 5) indicating very good reproducibility of the TOM tool.

### 3.4. Responsiveness (Sensitivity to Change)

According to the responsiveness evaluation, the TOM score significantly increased by an average of 4 points from the 3- to 6-month evaluation (Wald test; *P* < 0.001) and by 6 points from the 3- to 12-month evaluation (Wald test; *P* < 0.001) (Table 2). 

## 4. Discussion

Recent trends in clinical trial research have placed an increasing focus on understanding health outcomes from the patient's perspective [6]. This has led to the latest implementation of new regulations by the United Kingdom National Health Service and definitive guidelines on patient-reported outcomes set by the US Food and Drug Administration [4, 26]. The use of standardised outcome measures based on detailed information from the patient now plays an important factor when considering the primary treatment objectives of every new clinical trial. Our development of the TEFTOM questionnaire highlights this increasing requirement for outcome measures focusing on patient-rated assessments. Furthermore, the TEFTOM instrument can specifically be used to evaluate patient-rated expectations; this is of particular importance when considering that the average trauma patient—as opposed to those with chronic disease—cannot adequately provide a baseline score. 

Reliable indicators of healthcare quality are important to accurately measure performance and promote improvements in services [27]. In the context of trauma surgery, pretraumatic conditions are—in most of the cases—too high a benchmark for success. Previous research in this field has indicated that *informed* patient expectations for their surgery outcome may represent a valid means of assessing the quality of a surgical procedure following a fracture [11]. It was with this spirit that we developed the TEFTOM questionnaire. We believe that the TEF score—obtained after the orthopaedic surgeon has informed the patient of their individual condition, chances of recovery, and possible consequences of surgery—can reliably quantify expectations on outcome after surgery and thus produce an individual summary expectation factor to be used as a reference to evaluate the recovery process as well as the final outcome. It is with the TOM score that after surgery, a patient- and condition-specific indicators of the ability to fulfil those expectations can be produced. 

Being able to provide an individual measure of expectation fulfilment is the striking advantage of the TEFTOM questionnaire. Most outcome assessment tools base judgment solely on the observation of general average trends. 

The outcome instrument TOM had good criterion validity against the AAOS, a tool that aims at measuring a similar construct. We found a lower correlation with the FAOS tool; this may probably be explained by the fact that TOM summarises five dimensions in a single overall score, whereas FAOS considers five dimensions individually. The moderate correlations (i.e., 0.5 to <0.7) between the TOM instrument and the Physical Component Summary of the SF-36 throughout the 1-year testing period may be due to the fact that the dimensions covered by the TOM instrument are not equivalent to those covered by the general SF-36 questionnaire. Moreover, the aspect of injury satisfaction is a specific dimension of the TOM tool that is not available in the SF-36 questionnaire. The correlation between AAOS and SF-36 has been reported to be 0.65 [13] and is based on the unweighted mean of the three SF-36 physical subscales. This is similar to the correlation between TOM and the mean of the three SF-36 subscales measured in our study population. 

An additional advantage of the TOM tool over the AAOS is that missing item imputation is rarely required for the TOM tool. For instance, many patients could already provide complete answers to the TOM tool at the 3-month examination, while many patients who did not recover completely could not answer the complete set of questions of the AAOS tool. Moreover, a ceiling effect was observed with the AAOS, whereas the TOM tool was less inclined to be affected by an upper limit. These positive characteristics of the TOM tool indicate that it might be better in evaluating the healing process than the AAOS.

The limitations of this study include the focus of testing the TEFTOM questionnaire on trauma patients with isolated ankle and distal tibia fractures and using a Pan-American population. However, to obtain first-hand experience with this new measure, we decided to target a specific trauma condition. We believe TEFTOM is a promising general outcome measure that could also be adapted for use with nonoperative treatments. This cohort of patients, however, was not studied and should be the subject of future investigations. Cross-cultural adaptation testing is currently underway for TEFTOM to be used at an international level.

## 5. Conclusions

On the basis of this first validation study, TEFTOM proved to be valid, internally consistent, reproducible, and responsive to change in assessing the condition of ankle and distal tibia fracture patients after surgery. We believe that TEFTOM is a promising tool in measuring general trauma outcomes and performances. As an indicator of patient expectation fulfilment, this new measure might have powerful implications on the assessment of healthcare quality within the field of traumatology.

## Figures and Tables

**Table 1:** Patient baseline sociodemographic characteristics.

Characteristics	
Gender, number (%)	
Female	90 (44.8)
Male	111 (55.2)
Age (years)	
Mean (SD)	41 (15)
Median (range)	41 (18; 82)
Race, number (%)	
Caucasian	161 (80.1)
Other	40 (19.9)
Marital status, number (%)	
Married	95 (47.3)
Single (never married)	70 (34.8)
Divorced/widowed/separated	36 (17.9)
Highest qualification obtained, number (%)	
Primary school	30 (14.9)
High school	97 (48.3)
Undergraduate	50 (24.9)
Graduate/postgraduate	24 (11.9)
Employed, number (%)	
Yes	143 (71.1)
No	58 (28.9)
Annual household income, number (%)	
Up to $25,000	49 (25.3)
$25,001–$75,000	84 (43.3)
Higher than $75,000	61 (31.4)

SD: standard deviation; TEF: Trauma Expectation Factor; TOM: trauma outcome measure.

**Table 2:** Mean patient-reported scores for the TEFTOM, AAOS, FAOS, and SF-36 questionnaires.

		Followup						
Outcomes	*n*	Preoperative	Postoperative*	2 weeks	6 weeks	3 months	6 months	12 months
		Mean (95% CI)	Mean (95% CI)	Mean (95% CI)	Mean (95% CI)	Mean (95% CI)	Mean (95% CI)	Mean (95% CI)
TEF	201	33.9 (33.2; 34.6)	34.4 (33.6; 35.1)	34.1 (33.4; 34.9)	33.9 (33.1; 34.7)	34.9 (34.1; 35.6)	35.3 (34.5; 36.1)	
TOM	175					26.3 (25.4; 27.3)	30.4 (29.4; 31.4)	32.6 (31.6; 33. 6)
AAOS	174					72.3 (69.9; 74.7)	82.9 (80.4; 85.3)	89.0 (86.6; 91.3)
FAOS								
Symptoms	175					63.7 (60.7; 66.7)	71.4 (68.3; 74.5)	79.0 (76.0; 82.0)
Pain	175					72.9 (70.3; 75.5)	81.1 (78.4; 83.8)	86.1 (83.5; 88.7)
ADL	175					80.2 (77.9; 82.5)	89.8 (87.4; 92.2)	92.7 (90.4; 95.0)
Sport	173					43.5 (39.4; 47.6)	64.2 (60.0; 68.5)	76.7 (72.6; 80.7)
QOL	175					44.4 (40.9; 47.8)	55.3 (51.8; 58.9)	65.2 (61.9; 68.6)
SF-36 PCS	175					40.9 (39.4; 42.4)	47.1 (45.6; 48.6)	50.9 (49.4; 52.3)

AAOS: American Association for Orthopaedic Surgeons Foot and Ankle Score; ADL: activities of daily living; CI: confidence interval; FAOS: Foot and Ankle Outcome Score; PCS: Physical Component Summary; QOL: quality of life; SF-36: Short Form-36 Health Survey; TEF: trauma expectation factor; TOM: trauma outcome measure.

*Immediate postoperative examination.

**Table 3:** Correlation of the TOM portion of the questionnaire with the AAOS, FAOS, and SF-36 questionnaires.

	Followup					
	3 months		6 months		1 year	
	n	r	n	r	n	r
AAOS	142	0.77	134	0.76	155	0.69
FAOS						
Symptoms	151	0.61	134	0.58	156	0.52
Pain	152	0.70	134	0.62	156	0.62
ADL	152	0.69	133	0.59	156	0.57
Sport	146	0.49	133	0.53	154	0.52
QOL	152	0.64	134	0.64	156	0.63
SF-36 PCS	152	0.58	134	0.69	156	0.61
SF-36 PF, RP, and BP*	152	0.68	134	0.73	156	0.66

AAOS: American Association for Orthopaedic Surgeons Foot and Ankle Score; ADL: activities of daily living; BP: bodily pain; FAOS: Foot and Ankle Outcome Score; PF: physical function; PCS: Physical Component Summary; QOL: quality of life; *r*: Pearson's correlation coefficient; RP: role-physical; SF-36: Short Form-36 Health Survey; TEF: trauma expectation factor; TOM: trauma outcome measure.

*Unweighted mean of three SF-36 physical subscales (physical function, role-physical, and bodily pain).

**Table 4:** TEF internal consistency and reproducibility.

	Internal consistency		Test-retest reliability (reproducibility)	
	*n*	Cronbach's alpha	n*	ICC
Preoperative	200	0.69		—^†^
Postoperative	193	0.69	70	0.80
2 weeks	160	0.74	67	0.67
6 weeks	158	0.76	56	0.88
3 months	152	0.76	50	0.77
6 months	134	0.65	44	0.94

ICC: intraclass correlation coefficient; TEF: trauma expectation factor; TOM: trauma outcome measure.

*Number of subjects who volunteered to participate in the reliability assessment of the TEF questionnaire.

^†^No reliability assessment was performed on preoperative scores.

**Table 5:** TOM internal consistency and reproducibility.

	Internal consistency		Test-retest reliability (reproducibility)	
	*n*	Cronbach's alpha	n*	ICC
3 months	152	0.85	49	0.92
6 months	134	0.78	46	0.94
12 months	156	0.76	59	0.96

ICC: intraclass correlation coefficient; TEF: trauma expectation factor; TOM: trauma outcome measure.

*Number of subjects who volunteered to participate in the reliability assessment of the TOM questionnaire.

## Appendices

### A. Trauma Expectation Factor (TEF)

(1) One year (12 months) after surgery, how painful do you expect your injury to be?
  0 (No pain)
  1
  2
  3
  4 (Unbearable pain)
(2) One year (12 months) after surgery, how much do you expect your injury to interfere with your normal/usual *necessary* activity (including prolonged standing, walking, stairs, car driving, and sleeping)?
  0 (Not at all)
  1
  2
  3
  4 (Completely)
(3) One year (12 months) after surgery, how much do you expect your injury to interfere with your normal/usual *physical* activity (including work, housework, school, and recreation/sports activities)?
  0 (Not at all)
  1
  2
  3
  4 (Completely)
(4) One year (12 months) after surgery, how much do you expect your injury to interfere with your normal/usual *activities of daily living* (including eating, dressing, putting on shoe wear)?
  0 (Not at all)
  1
  2
  3
  4 (Completely)
(5) One year (12 months) after surgery, how much do you expect your injury to interfere with your normal/usual *relationships* (including family, friends, and coworkers)?
  0 (Not at all)
  1
  2
  3
  4 (Completely)
(6) Necessary activities (you have to do these). One year (12 months) after surgery, how much do you expect to cut down on the physical activities you have to do (including work, housework, and school)?
  0 (0%)
  1 (25%)
  2 (50%)
  3 (75%)
  4 (100%)
(7) Optional activities (you enjoy to do these). One year (12 months) after surgery, how much to you expect to cut down on the physical activities you enjoy doing (including sports, recreation, gardening, etc.)?
  0 (0%)
  1 (25%)
  2 (50%)
  3 (75%)
  4 (100%)
(8) One year (12 months) after surgery, how satisfied do you expect to be with your pain, physical function, and disability?
  0 (Not satisfied)
  1
  2
  3
  4 (Very satisfied)
(9) One year (12 months) after surgery, how satisfied do you expect to be with the appearance of your injury?
  0 (Not satisfied)
  1
  2
  3
  4 (Very satisfied)
(10) One year (12 months) after surgery, how satisfied do you expect to be with your overall well being?
  0 (Not satisfied)
  1
  2
  3
  4 (Very satisfied)

### B. Trauma Outcomes Measure (TOM) Questionnaire

(1) How painful is your injury today?
  0 (No pain)
  1
  2
  3
  4 (Unbearable pain)
(2) How much does your injury currently interfere with your normal/usual *necessary* activity (including prolonged standing, walking, stairs, car driving, and sleeping)?
  0 (Not at all)
  1
  2
  3
  4 (Completely)
(3) How much does your injury currently interfere with your normal/usual *physical* activity (including work, housework, school, and recreational/sports activities)?
  0 (Not at all)
  1
  2
  3
  4 (Completely)
(4) How much does your injury currently interfere with your normal/usual *activities of daily living* (including eating, dressing, and putting on shoe wear)?
  0 (Not at all)
  1
  2
  3
  4 (Completely)
(5) How much does your injury currently interfere with your normal/usual *relationships* (including family, friends, and coworkers)?
  0 (Not at all)
  1
  2
  3
  4 (Completely)
(6) Necessary activities (you have to do these). How much do you currently cut down on the physical activities you have to do (including work, housework, and school)?
  0 (0%)
  1 (25%)
  2 (50%)
  3 (75%)
  4 (100%)
(7) Optional activities (you enjoy to do these). How much do you currently cut down on the physical activities you enjoy doing (including sports, recreation, and gardening)?
  0 (0%)
  1 (25%)
  2 (50%)
  3 (75%)
  4 (100%)
(8) How satisfied are you with your current level of pain, physical function, and disability?
  0 (Not satisfied)
  1
  2
  3
  4 (Very satisfied)
(9) How satisfied are you with the current appearance of your injury?
  0 (Not satisfied)
  1
  2
  3
  4 (Very satisfied)
(10) How satisfied are you with your current overall well being?
  0 (Not satisfied)
  1
  2
  3
  4 (Very satisfied).

## Abbreviations

AAOS: American Academy of Orthopaedic Surgeons Foot and Ankle Scale
AO/OTA: AO Foundation and Orthopaedic Trauma Association
FAOS: Foot and Ankle Outcome Score
ICC: Intraclass coefficient correlation
SF-36: Short Form-36 Health Survey
TEFTOM: Trauma Expectation Factor Trauma Outcome Measure
US: United States.
